# Can We Use an Interventionist-Causal Paradigm to Untangle the Relationship between Trauma, PTSD and Psychosis?

**DOI:** 10.3389/fpsyg.2017.00306

**Published:** 2017-03-03

**Authors:** Rachel M. Brand, Susan L. Rossell, Sarah Bendall, Neil Thomas

**Affiliations:** ^1^Centre for Mental Health, Swinburne UniversityHawthorn, VIC, Australia; ^2^The Voices Clinic, Monash Alfred Psychiatry Research Centre, Alfred Hospital and Monash University Central Clinical SchoolMelbourne, VIC, Australia; ^3^Psychiatry, St. Vincent's HospitalFitzroy, VIC, Australia; ^4^Orygen: The National Centre of Excellence in Youth Mental HealthParkville, VIC, Australia; ^5^The Centre for Youth Mental Health, The University of MelbourneParkville, VIC, Australia

**Keywords:** trauma, psychosis, posttraumatic stress disorder, schizophrenia, causal, methodology

There is mounting evidence that exposure to traumatic or adverse life-events is associated with increased risk of psychosis (Read et al., 2001; Bendall et al., 2008, 2010; Read and Bentall, 2012). However, to inform treatment and prevention, it is necessary to go beyond association to understand *how* traumatic experiences may lead to the development of psychotic symptoms. In this paper, we argue that doing so requires the identification of biological, psychological and social processes that may be involved in the observed trauma–psychosis relationship, and determining which are causally related. We propose that this can be done in conjunction with focused intervention procedures that may test theoretical mechanisms, in parallel with piloting potential components of therapeutic interventions.

A recent proliferation of research has examined a broad range of factors as putative causal mechanisms. One important strand of this research has drawn on the particular relationship between trauma, posttraumatic stress disorder (PTSD) and psychosis. PTSD is one of the most rigorously researched sequelae of trauma exposure and is, by definition, caused by traumatic events. There are high rates of comorbidity between PTSD and psychosis (Kilcommons and Morrison, 2005; Sareen et al., 2005; Anketell et al., 2010), and PTSD is a risk factor in the subsequent development of psychosis (Okkels et al., 2017). This relationship may provide an insight into the mechanisms through which trauma exposure can lead to the emergence and maintenance of psychosis.

To make causal inferences regarding these putative mechanisms, the literature needs to move beyond establishing association to experimental studies in which trauma exposure, PTSD symptoms or causal mechanisms involved in PTSD, are subject to controlled manipulations. However, there are feasibility and ethical issues in this undertaking and we therefore propose that a research paradigm referred to as the “interventionist-causal” approach offers a critical way forward. We pay particular attention to trauma-related psychological mechanisms, with a view that a more sophisticated understanding of the causal role of these mechanisms will lead to much needed improvements in psychological interventions for psychosis (Freeman, 2011; Thomas et al., 2014). While identifying causal mechanisms is not the only way of addressing recovery, this process of intervention development can add value to broader intervention approaches. Indeed, this process has been helpful in refining and improving the efficacy of psychological interventions for anxiety (Clark, 2004).

## Mechanisms linking PTSD and psychosis

Mueser et al. (2002) proposed that PTSD symptomatology itself mediates the relationship between trauma exposure and the course of serious mental illness, particularly schizophrenia. Whilst not commenting on whether PTSD plays a casual role in the development of psychosis, this theory places PTSD symptomatology centrally in understanding the exacerbation of psychotic symptoms.

Morrison (2003) went further, proposing that rather than being separate, psychotic symptoms and PTSD fall on a continuum of trauma-related reactions and are caused and maintained by similar psychological mechanisms. Researchers have since further elucidated psychological mechanisms involved in specific symptoms of psychosis that may be shared with those involved in PTSD. The correlation between posttraumatic intrusions and hallucinations in trauma-affected populations (Gracie et al., 2007; Alsawy et al., 2015; Ayub et al., 2015) and the fact that the content of hallucinations often have thematic or direct links with trauma content (Read et al., 2003; Hardy et al., 2005; Corstens and Longden, 2013; McCarthy-Jones et al., 2014) has led to the proposal that some hallucinations may in fact be a form of posttraumatic intrusion. Contemporary psychological theories of PTSD conceptualize the nature of cognitive processing during traumatic events to be central to the development of posttraumatic intrusions (Ehlers and Clark, 2000; Brewin, 2001; Brewin et al., 2010). Shifts in information-processing style during traumatic events are posited to lead to trauma memories that are de-contextualized, fragmented, dominated by sensory information, and sensitive to involuntary priming. The nature of cognitive processing during traumatic events has also been implicated in the development of hallucinations in the general population (Geddes et al., 2016) and in people high in schizotypy (Steel et al., 2005).

Dissociation, another psychological process implicated in PTSD, has also been linked to hallucinations following trauma. Indeed, many researchers propose that hallucinations are dissociative phenomena (Moskowitz and Corstens, 2007; Longden et al., 2012). Dissociation is correlated with hallucinatory experiences (Pilton et al., 2015), mediates the relationship between childhood trauma and hallucinations (Perona-Garcelán et al., 2012; Varese et al., 2012) and predicts hallucinations in the flow of daily life (Varese et al., 2011).

Hallucinations are not the only psychotic symptom that has been linked to PTSD symptoms and related mechanisms. Associations between delusional beliefs and PTSD symptoms have been observed following traumatic events (Freeman et al., 2011; Ayub et al., 2015) and the same cognitive factors have been found to predict both paranoia and PTSD following a physical assault (Freeman et al., 2013). Research has been more equivocal with regards to negative symptoms (Lysaker and Larocco, 2008; Strauss et al., 2011) but it has been suggested that these can be manifestations of the avoidance of traumatic memories (Stampfer, 1990; McGorry, 1991; Morrison, 2003).

In summary, there is evidence of a close relationship between PTSD and psychotic experiences, but questions remain regarding whether PTSD symptomatology itself represents a casual mechanism in the development or maintenance of psychosis, or whether shared mechanisms underpin the causal relationships between trauma and both PTSD and psychosis outcomes. Establishing causal inferences regarding psychological mechanisms involved in psychosis is, of course, complex. Symptoms are likely to be caused by multiple mechanisms and each mechanism is likely only to contribute to the probability of a symptom occurring. Nonetheless, identifying the potential role of each PTSD-related psychological mechanism in the development and maintenance of psychosis will inform more evidence-driven and targeted psychological interventions for trauma-related psychoses. A particularly tantalizing aspect of the relationship between PTSD and psychosis is that there are already well-established, effective treatments for PTSD. If psychological mechanisms involved in PTSD do play a causal role in psychotic experiences, this would open up promising new treatments for psychotic symptoms.

## Beyond association to identifying causal mechanisms

In order to establish the causal role of candidate mechanisms, certain criteria must be met. Despite a lack of consensus on the precise definition of causality, epidemiologists have outlined the essential properties of causal relationships; namely, that there is an association between the variables, that the cause temporally precedes the effect, that change in the putative causal variable leads to change in the outcome, and that spurious, confounding variables in this relationship are controlled for (also referred to as sole plausibility) (Reininghaus et al., 2016).

Thus far, research in the field has predominantly involved cross-sectional studies that examine associations between trauma, PTSD, psychosis, and putative shared mechanisms. Cross-sectional studies are, however, limited in drawing causal inferences, since it is not possible to robustly establish temporal relationships and sole plausibility. There are also examples of prospective studies in the area, which have built on these cross-sectional associations by establishing temporal ordering (Okkels et al., 2017). However, prospective studies can be time intensive and still do not offer control over extraneous variables to establish sole plausibility. Observational studies that observe natural fluctuations in putative mechanisms and how these interact with symptoms have also made recent valuable additions to the literature, particularly with the use of mobile technology in ecological momentary assessment studies (e.g., Varese et al., 2011). Yet, without controlled manipulation of variables it is again difficult to establish sole plausibility (Reininghaus et al., 2016).

We argue that what is now needed are experimental approaches using controlled manipulations of trauma exposure, PTSD symptoms, or putative shared casual mechanisms and an assessment of the impact of these manipulations on psychotic symptoms. There have been initial examples of this in the use of the analog trauma paradigm, in which trauma exposure is experimentally manipulated (with the presentation of a distressing film) and outcomes examined (e.g., Marks et al., 2012). There are, however, challenges in the design and execution of these studies in trauma-affected and psychosis populations, possibly explaining why the majority of these studies have thus far been conducted with non-clinical samples. In clinical groups, there are ethical and clinical issues with introducing trauma exposure as an independent variable, or with inducing controlled increases in PTSD symptoms.

## An interventionist-casual paradigm for the investigation of the relationship between trauma, PTSD and psychosis

We propose that an alternative experimental model that holds promise in moving past this methodological impasse is the interventionist-causal paradigm. In this approach, causation is substantiated by controlled manipulation of the hypothesized causal mechanism and examination of the subsequent effect on the symptom of interest (Kendler and Campbell, 2009). In psychiatry research this can be accomplished using interventions proposed to act on causal mechanisms, establishing their effect on these mechanisms when compared with a control intervention to minimize other confounding variables, and observing the impact on the symptoms of interest. If this chain of causality can be established, then causal inferences regarding the mechanisms in question may be confirmed. In practice, this looks like a randomized controlled trial of an intervention, but as well as establishing treatment efficacy, we use this paradigm to further our understanding of causal mechanisms. An interventionist-causal paradigm has been previously noted for its use in understanding causal mechanisms in psychosis (Freeman, 2011; Garety and Freeman, 2013; Reininghaus et al., 2016). An attractive aspect of this model is that the experimental intervention is one that is designed to reduce problematic causal processes and thus (hypothetically) improve symptom outcomes of interest. This is well aligned with the ethos of the fields of clinical psychology and psychiatry.

The well-developed PTSD treatment literature gives us a head start in terms of assessing the causal role of PTSD symptomatology itself in psychotic experiences using the interventionist-causal model. Treatments that are already known to be effective in reducing PTSD symptoms, such as prolonged exposure, trauma focused CBT and Eye Movement Desensitization and Reprocessing therapy (EMDR) (Bisson et al., 2007), can be delivered to people with psychosis in controlled studies and the effects on both PTSD symptoms and psychotic symptoms established. Approaches to date using these treatments for people experiencing psychosis have focused on treating comorbid PTSD symptoms, demonstrating the safety of using such interventions and some positive effects on PTSD symptoms, particularly for EMDR and prolonged exposure (van den Berg et al., 2015), but less so for cognitive restructuring (Steel et al., 2017). There is, however, limited data on the impact of these interventions on psychotic symptoms.

Additionally, the putative shared mechanisms involved in both PTSD and psychosis can be subject to interventionist-causal enquiry using specific components of psychological interventions, ascertaining that they act on a mechanism of interest, and observing the effect on psychotic symptoms. This is somewhat more complex, since literature regarding the mechanisms of action of psychological treatments remains in its infancy, however the interventionist-causal model is well placed to deal with this complexity in separating specific mechanisms of action and their relationship to treatment outcomes. A promising example of this, a pilot trial of a brief CBT intervention for depersonalization in psychosis, is currently underway (Farrelly et al., 2016). We propose that this paradigm now needs to be extended to the multitude of other potential casual mechanisms implicated in PTSD and psychosis. Table 1 outlines interventions or intervention components that may be explored in an interventionist-causal model to explicate the casual role of these mechanisms.

**Table 1:** **Putative causal mechanisms involved in both PTSD and psychosis and interventions with which the interventionist-causal paradigm can be used to examine causality**.

**Putative mechanism**	**Intervention**
Trauma memory processing	Imaginal exposure, EMDR
Negative posttraumatic beliefs	Trauma-focused cognitive therapy, cognitive processing therapy
Dissociation	Cognitive behavioral interventions for dissociation
Posttraumatic avoidance	*In vivo* and imaginal exposure

In summary, we believe that interventionist-causal models offer a crucial next step in untangling the relationship between trauma, PTSD and psychosis. Importantly, the paradigm offers a way of extending our understanding beyond that of association, into establishing causal inferences. In addition, research of this nature can establish individual treatment components, acting on specific causal mechanisms, which can effectively be used to treat psychotic experiences in those who have experienced trauma.

## Author contributions

RB drafted the original manuscript and conceived of the idea for the content. NT contributed to conception of the ideas in the article and revised the manuscript. SB and SR revised the final manuscript and advised on content.

## Funding

The first author is supported by a Swinburne University Postgraduate Research Award (SUPRA).

### Conflict of interest statement

The authors declare that the research was conducted in the absence of any commercial or financial relationships that could be construed as a potential conflict of interest.
